# PRIMARY OUTCOMES FROM CAREGIVER-REPORTED SURVEYS IN THE SHARE TRIAL

**DOI:** 10.1093/geroni/igad104.0040

**Published:** 2023-12-21

**Authors:** Jennifer Wolff, Erin Giovannetti, Cynthia Boyd, David Roth, Talan Zhang, Diane Echavarria, Valecia Hanna, Daniel Scerpella

**Affiliations:** Johns Hopkins University, Baltimore, Maryland, United States; MedStar Health Research Institute, Baltimore, Maryland, United States; Johns Hopkins School of Medicine, Baltimore, Maryland, United States; Johns Hopkins University, Baltimore, Maryland, United States; Johns Hopkins School of Medicine, Baltimore, Maryland, United States; Johns Hopkins University, Baltimore, Maryland, United States; Johns Hopkins School of Public Health, Baltimore, Maryland, United States; Johns Hopkins Bloomberg School of Public Health, Baltimore, Maryland, United States

## Abstract

This presentation will introduce SHARE, a randomized-controlled trial to proactively engage family caregivers and normalize advance care planning in primary care. The objective of the study is to engage family caregivers in longitudinal interactions with primary care clinicians and stimulate and support ACP discussions in primary care. We focus on persons with mild, moderate, and severe cognitive impairment regardless of clinical diagnosis because of the importance of addressing advance care planning early in the disease trajectory, the under-diagnosis of ADRD, and because of the greater implementation potential of a protocol with broader applicability. We will describe the study design and present on 6-month outcomes to test our hypothesis that as compared with the minimally enhanced control care group, caregivers in the intervention group will report better quality of communication about end-of-life care with primary care clinicians at 6 months (primary outcome).

